# Combined SPME—GC × GC–MS Volatilomics and UHPLC–HRMS Metabolomics Data Fusion Provides a Multidimensional Fingerprinting and Discrimination of White Garlic From the Piacenza Region

**DOI:** 10.1111/1750-3841.70601

**Published:** 2025-10-05

**Authors:** Giulia Leni, Marco Armando De Gregorio, Elena Secomandi, Leilei Zhang, Terenzio Bertuzzi, Marco Trevisan, Giorgia Spigno, Luigi Lucini

**Affiliations:** ^1^ Department for Sustainable Food Process Università Cattolica del Sacro Cuore Piacenza Italy; ^2^ Department of Science, Technology and Society Scuola Universitaria Superiore IUSS Pavia Pavia Italy; ^3^ Department of Animal Science, Food and Nutrition Università Cattolica del Sacro Cuore Piacenza Italy

**Keywords:** *Allium sativum* | data fusion | garlic quality | multi‐omics

## Abstract

Garlic quality is attributed to a diverse array of volatile and non‐volatile compounds, and several varieties are protected under geographical indication schemes, whereas many typical local cultivars remain chemically uncharacterized. Among them, white garlic from Piacenza (Italy) is a traditional product with recognized sensory attributes. In this study, a comprehensive characterization of the volatile and non‐volatile profiles of this variety was performed, followed by its discrimination against other European garlic cultivars. To achieve this, an integrated foodomics approach, based on both ultrahigh‐performance liquid chromatography coupled with high‐resolution mass spectrometry (UHPLC–HRMS) and solid‐phase microextraction—bi‐dimensional gas chromatography coupled with mass spectrometry (SPME–GC × GC–MS) analyses, was applied, along with chemometric modeling and data fusion with a DIABLO framework. A total of 89 volatile and over 2300 non‐volatile compounds were identified. Piacenza garlic exhibited a significantly higher accumulation of aldehydes and revealed a diversity of organosulfur compounds, polyphenols, dipeptides, and flavonoids. Chemometrics highlighted the presence of chemical clusters that distinguished the Piacenza variety from the others. These findings were also confirmed by the data fusion approach, which identified specific aldehydes, sulfur, and phenolic compounds as key discriminant molecules. This comprehensive chemical fingerprinting supports the valorization of Piacenza white garlic. In addition, the proposed approach provides a valuable tool for the characterization and authentication of agri‐food products.

**Abbreviations**
^1^H NMR: proton nuclear magnetic resonance
AUC: area under the ROC curve
HCA: hierarchical cluster analysis
LC–MS/MS: liquid chromatography–tandem mass spectrometry
LOD: limit of detection
LRI: linear retention index
OPLS‐DA: orthogonal partial least squares discriminant analysis
PCA: principal component analysis
PDO: protected designation of origin
PGI: protected geographical indication
PLS‐DA: partial least squares discriminant analysis
ROC: receiver operating characteristic
sGCCA: generalized canonical correlation analysis
SPME–GC × GC–MS: solid‐phase microextraction—bi‐dimensional gas chromatography coupled with mass spectrometry
UHPLC–HRMS: ultrahigh‐performance liquid chromatography coupled with high‐resolution mass spectrometry
VIP: variable importance in projection

## 1 Introduction

Nowadays, over 28 million tonnes of garlic (*Allium sativum* L.) are produced globally, making it a significant commercial food crop (FAO 2025). Garlic is mainly used for gastronomy purposes and is commercially present in different forms, including bulbs, flakes, powder, and essential oil (Martins et al. 2016). In addition, it is considered among the oldest and most referenced herbs involved in therapeutic formulas for treating and preventing many disorders (Fenwick and Hanley 2009). Among the biological activities recognized in garlic, it can be mentioned that it has anti‐obesity, cardioprotective, antihypertensive, antioxidant, and cholesterol‐lowering properties (Javed and Ahmed 2022). These valuable properties are ascribable to several bioactive molecules, including sulfur‐containing molecules, responsible for the typical garlic aroma. The presence of these compounds, and in general the chemical composition of garlic, is strictly connected and influenced by the genotype, the conditions, and the growing location (Martins et al. 2016). Nowadays, more than 1400 food products are registered and protected by geographical indications in the European Union, with 10 different garlic products included in the list. These varieties are Ail Violet de Cadours, Aglio Bianco Polesano, Ail de la Drôme, Ail Rose de Lautrec, Ajo Morado de Las Pedroñeras, Ail fumé d'Arleux, Czosnek galicyjski, Ail Blanc de Lomagne, Alho da Graciosa, and Aglio di Voghiera (European Commission 2025). Next to these, for which registered geographical indication schemes protect the name and the production process, a plethora of other garlic varieties exist, the production of which is strictly connected to a geographical area but are not yet included in this list.

In this context, analytical tools able to verify and trace the origin of a food product became of primary importance due to the close connection between food quality and authenticity (Li et al. 2020). Stable isotope analysis combined with pairwise partial least squares discriminant analysis (PLS‐DA) models was demonstrated useful in the discrimination of Jinxiang garlic (with protected geographical indication [PGI] status in China and the European Union) from varieties produced by other significant exporting nations (i.e., Vietnam and Argentina, Thailand and Southeast Asian countries) (Nie et al. 2021). Furthermore, Nie et al. (2022) demonstrated the potential of stable isotopes, combined with targeted analysis of sulfur‐containing compounds performed with liquid chromatography–tandem mass spectrometry (LC–MS/MS) and chemometric analysis, for discriminating the geographical origins of Chinese garlic varieties. An untargeted metabolomics approach was performed by Leite et al. (2024) with gas chromatography coupled with MS (GC–MS) to reveal through chemometrics the distinct metabolite signatures of Brazilian and Chinese garlic varieties. Molina‐Calle et al. 2016. performed a combination of headspace analysis with GC–MS to detect and distinguish the volatile profile of three garlic varieties cultivated in the South of Spain (2016). Additionally, untargeted metabolomics performed with Fourier‐transform ion cyclotron resonance MS was demonstrated useful for assessing the distinct distribution of CHO and CHNO molecules in eight *Allium* bulbs from the wild *A. triquetrum*, *A. roseum*, and *A. ampeloprasum* species as well as the cultivated *A. sativum* (Maccelli et al. 2020). Forty‐seven samples of garlic from China, Spain, and the Czech Republic were subjected to untargeted metabolomic analysis by using three different high‐resolution MS (HRMS) instruments (Hrbek et al. 2018). Furthermore, it was demonstrated that the high‐resolution ^1^H NMR method, in combination with multivariate statistics, was effective in discriminating Chinese garlic from the Korean variety (Jo et al. 2020) and in classifying Italian samples according to their cultivars (Ritota et al. 2012).

In the present work, white garlic from Piacenza, a typical Italian agri‐food product recognized by the Emilia Romagna Region and produced in a specific area in the province of Piacenza, was analyzed as a case study to highlight the differences with other European garlic varieties. This variety is known for the compact bulbs with a pungent flavor and white, dry outer tunics, as well as a prolonged shelf‐life, making it highly valued by consumers (Emilia Romagna Region 2025). Despite its regional recognition and sensory characteristics, this variety is not currently included in the list under the geographical indication schemes of the European Union. Given the increasing emphasis on traceability and authenticity in the food sector, scientifically characterizing its phytochemical and volatile profile is essential to support its valorization and potential inclusion in protected designation frameworks, also contributing to the regional economy. To our knowledge, only Ritota et al. (2012) characterized this variety using HRMAS‐NMR spectroscopy and solid‐phase microextraction (SPME)–GC–MS, identifying chemical differences with three other Italian varieties of red garlic. However, only a limited number of metabolites were identified through both analytical techniques, underlying the need for a more in‐depth characterization to depict the complex profile of this product. To achieve a comprehensive profile of this garlic, foodomics approaches, combining both metabolomic and volatilomic strategies, were integrated for the first time to characterize the phytochemical and volatile profile of the different garlic samples. Given the complementarity of the approaches proposed, the hypothesis underlying our work is that data fusion may provide a comprehensive signature of garlic, thus supporting its quality and authenticity.

## 2 Materials and Methods

### 2.1 Samples

Ten different varieties of garlic (*A. sativum* L.) were used for the study: red garlic from Molise (Italy, code G1), PDO Polesano white garlic (Italy, code G2), Ajo Morado de Las Pedroñeras PGI (Spain, code G3), white garlic from Piacenza (Pallavicino variety, Italy, code G4), Nubia red garlic (Italy, code G5), PDO Voghera garlic (Italy, code G6), Sulmona red garlic (Italy, code G7), Garcua white garlic (Spain, code G8), white garlic from Piacenza (Monticelli variety, Italy, code G9), and Ail Rose de Lautrec PGI (France, code G10).

Five bulbs, from independent field sites, were made available for each sample by the “Cooperativa Produttori Aglio Bianco Piacentino” farmers association (Piacenza, Italy) or from local markets. Fresh bulbs, after manually peeling, were pooled and minced using a laboratory mill (MX67, Girmi, Rimini, Italy) for 1 min in an ice bath. One gram of sample was placed into a 20‐mL headspace vial for volatile profiling, whereas the remaining was immediately stored at −20°C until metabolomic analysis.

### 2.2 SPME–GC × GC–MS Analysis of Volatiles

The analysis was carried out in triplicate with SPME–GC × GC–MS instrument (GCMS‐TQ8040 NX, Shimadzu, Kyoto, Japan) equipped with a flow modulator, according to Leni et al. (2024). The samples were equilibrated at 40°C for 20 min under agitation before the volatile extraction. The headspace was sampled at 40°C for 20 min with a wide range of carbon/polydimethylsiloxane (PDMS) fiber (Shimadzu, Kyoto, Japan). The desorption was performed at 260°C for 5 min into the GC injection port. The analysis was performed in splitless injection mode. The column configuration was first‐dimension SLB‐5 ms fused silica capillary column (20 m × 0.18 mm × 0.18 µm film thickness, Supelco) coupled to a second‐dimension SupelcoWAX (5 m × 0.32 mm × 0.25 µm film thickness, Supelco). The flow modulator was set at 4 s. Carrier gas was helium with 82 kPa as column pressure, 0.6 mL/min as column flow, and 34.1 cm/s as linear velocity. The oven temperature was programmed to hold at 40°C for 2 min, then ramped to 220°C at a rate of 6°C/min. The additional flow controller (APC) was held for 2 min at a pressure of 4.5 kPa, then was increased to 54.6 kPa at 1.67 kPa/min. Mass spectra were acquired in scan mode (*m*/*z* range of 50–650), an event time of 0.03 s, and a scan speed of 20,000 as. The ion source was set at 200°C and the MS interface at 220°C. The two‐dimensional chromatograms were processed (eliminate noise peaks and identify peaks with signal‐to‐noise ratios exceeding 100) with Shimadzu LabSolutions—Post Run software (Shimadzu, Kyoto, Japan). Peaks were identified by (i) matching the acquired mass spectra with the NIST20 database and (ii) comparing the calculated linear retention index (LRI) with literature values (NIST Chemistry WebBook). LRI was calculated after running a series of alkanes (C7–C20, Sigma Aldrich) on the same chromatographic conditions as the samples, considering the retention time on the first column. Compounds were considered identified when their mass spectra showed a match of at least 80% with entries in the NIST20 database, and the LRI differed by no more than 10 U from the literature value (NIST Chemistry WebBook) reported for columns of the same polarity. Compounds were considered tentatively identified when only the mass spectral match met the 80% similarity threshold (Dias et al. 2021). The relative abundance of compounds was expressed as the base‐10 logarithm of each compound's total integrated area, normalized to the sample's fresh weight.

### 2.3 Fingerprinting by Untargeted Metabolomics Based on Ultrahigh‐Performance Liquid Chromatography Coupled With High‐Resolution Mass Spectrometry (UHPLC–HRMS)

One gram of minced sample was accurately weighed and extracted using 10 mL of a hydroalcoholic solution composed of 80% methanol acidified with 0.1% formic acid (v/v). Extraction was carried out using a homogenizer (Politron, IKA T10, Staufen, Germany) for 2 min at room temperature. The mixture was subsequently centrifuged at 7800 × *g* for 15 min at 4°C (Eppendorf 5410R, Hamburg, Germany). An aliquot of 1 mL of the resulting supernatant was filtered through a 0.22 µm cellulose membrane and transferred into a 2 mL autosampler vial. Five biological replicates were prepared for each of the 10 garlic cultivars analyzed. A pooled quality control (QC) sample was generated by combining 30 µL of each individual extract.

Untargeted metabolomic profiling was performed using a Q Exactive Focus hybrid quadrupole‐Orbitrap MS (Thermo Fisher Scientific, Waltham, MA, USA) coupled to a Vanquish UHPLC system equipped with a heated electrospray ionization (HESI) source (Thermo Fisher Scientific, USA). Chromatographic separation conditions were optimized according to Becchi et al. (2023). Analyses were carried out using a reverse‐phase C18 column (Agilent Zorbax Eclipse Plus, 4.6 mm × 150 mm, 160 m^2^/g surface area, 95 Å pore size) and a linear gradient elution of water and acetonitrile ranging from 6% to 94% over 33 min at a flow rate of 200 µL/min. Full‐scan MS data were acquired in positive ionization mode across an *m*/*z* range of 100–1200 at a resolution of 70,000 FWHM (full width at half maximum) at *m*/*z* 200. The injection volume was 6 µL, with an automatic gain control (AGC) target of 10^6^ and a maximum injection time (IT) of 200 ms. QC samples were injected in a randomized sequence. Data‐dependent MS/MS acquisition (Top *N* = 3) was conducted in full‐scan mode (*m*/*z* 200–17,500), using an AGC target of 10^5^, maximum IT of 100 ms, and an isolation window of 1.0 *m*/*z*. Fragmentation of precursor ions was performed using stepped normalized collision energies of 10, 20, and 40 eV. HESI source parameters were as follows: sheath gas flow 40 arbitrary units (arb), auxiliary gas flow 20 arb, spray voltage 3.5 kV, and capillary temperature 320°C.

Raw UHPLC–HRMS data were processed using the open‐source MS‐DIAL software (version 4.9). The workflow included peak detection, feature alignment, and compound annotation via spectral library matching. Raw files (.raw) were converted to .abf format using the Reifycs ABF Converter. Feature extraction was carried out within the 100–1200 *m*/*z* range, using a minimum peak height of 3000 counts and a retention time window of 1–32 min. Tolerances were set at 0.01 Da for MS and 0.05 Da for MS/MS centroiding. Compound identification was based on exact mass accuracy, isotopic pattern, and MS/MS spectral similarity using the FooDB database (https://foodb.ca), applying a minimum identification score of >80%. During alignment, features present in fewer than 75% of replicates within each group were excluded. Final annotation was based on a total identification score threshold of >60%, accounting for common HESI‐positive adducts. Gap‐filling was performed using a peak‐finder algorithm with a mass tolerance of 5 ppm. Annotations were reported with Level 2 confidence (putatively annotated compounds), in accordance with the COSMOS Metabolomics Standards Initiative (Salek et al. 2015).

### 2.4 Statistical Analysis

#### 2.4.1 Volatilomics Dataset

The volatilomics dataset was elaborated by MetaboAnalyst 5.0. Missing values were imputed by substituting them with LOD, calculated as one‐fifth of the smallest positive value of each variable. Subsequently, median normalization was performed on the dataset, followed by logarithmic transformation and auto‐scaling. ANOVA followed by Tukey as a post hoc test (SPSS v26.0, Chicago, IL, USA) was performed to determine significant differences among volatile chemical groups (*p* < 0.05). Then, the volatilomics dataset was subjected to principal component analysis (PCA) and orthogonal partial least squares discriminant analysis (OPLS‐DA) to explore sample similarities and differences among garlic varieties (MetaboAnalyst 5.0). Variable importance in projection (VIP) scores was used to assess the contribution of individual volatile compounds to sample discrimination, with a threshold set at VIP >1 (MetaboAnalyst 5.0).

#### 2.4.2 Metabolomics Dataset

Metabolomic data were analyzed via an unsupervised approach by MetaboAnalyst 5.0 (Chong et al. 2019) and a supervised statistical analysis by SIMCA 16 (Umetrics, Malmo, Sweden). An unsupervised approach was employed to perform hierarchical cluster analysis (HCA), whereas a volcano plot analysis was conducted by integrating fold‐change (FC) evaluation (cut‐off > 1.5) with one‐way ANOVA (*p* < 0.05), followed by Tukey's HSD post hoc test and Bonferroni correction for multiple comparisons (family‐wise error rate). The OPLS‐DA model was performed as a supervised analysis, and the outcoming model was then validated by calculating the goodness of fit (*R* ^2^ *Y*) and the goodness of prediction (*Q* ^2^ *Y*). Furthermore, the model was checked for outliers (Hotelling's test) and model overfitting (permutation testing; *N* >100). To evaluate the importance of each metabolite in discriminating between different groups, the VIPs were calculated, and only compounds with a minimum significant threshold higher than 1.2 were considered. The log fold change analysis was calculated considering the pairwise comparison between the two varieties of white garlic from Piacenza and others. Receiver operating characteristic (ROC) curves were generated using MetaboAnalyst 5.0 to further assess the discriminative power of the identified variables based on VIP scores. The area under the ROC curve (AUC) was examined to evaluate the overall predictive performance of each discriminant compound. In addition, chemical similarity enrichment analysis (ChemRICH) was carried out using the ChemRICH online platform (accessed March 14, 2024), following the approach described by Barupal and Fiehn (2017).

#### 2.4.3 Data Fusion

A multi‐omics data integration analysis was conducted using SPME–GC × GC–MS and UHPLC–HRMS datasets to identify discriminant features distinguishing Piacenza's white garlic from other cultivars. The analysis was performed in R (version 4.1.3) using the *mixOmics* package suite. Integration was achieved through a supervised multivariate approach based on sparse partial least squares discriminant analysis (sPLS‐DA), implemented within the sparse generalized canonical correlation analysis (sGCCA) framework described by González et al. (2012), as part of the DIABLO pipeline. Feature selection across omics layers was performed by applying *ℓ*1 penalization to the variable coefficient vectors, in line with the method proposed by Tibshirani (1996). The final integrative model was constructed using two principal components, incorporating 100 and 40 features for UHPLC–HRMS data and 90 and 50 features for SPME–GC × GC–MS data, respectively. These are the numbers of metabolites selected from each component, derived from the stratified cross‐validation (3‐fold CV, 50 repetitions), which compares model performance under varying *ℓ*1 penalty parameters to achieve sparsity of the PLS‐DA. The optimal number of components was selected on the basis of the balanced error rate (BER).

## 3 Results and Discussion

### 3.1 Volatile Profile of Different Garlic Varieties

The comparative profile of the volatile compounds identified in different garlic varieties is reported in Table S1. Overall, 89 different molecules were detected by SPME–GC × GC–MS, with diallyl disulfide and (*E*)‐1‐allyl‐2‐(prop‐1‐en‐1‐yl)disulfane representing the most abundant volatile molecules detected in all samples (Table S1). The identified compounds belonged to the following chemical classes: acids, alcohols, aldehydes, alkanes, alkenes, aromatic compounds, ketones, *S*‐alk(en)yl‐l‐cysteine sulfoxide derivatives, and others. Significant differences (*p* < 0.05) were identified between the relative abundance of these chemical families across the different garlic varieties (Table 1).

**TABLE 1:** Changes in the total abundance (log_10_ of area) of volatile compound classes identified in the different garlic samples.

Chemical family	Red garlic from Molise	Polesano white garlic PDO	Ajo Morado de Las Pedroñeras PGI	White garlic from Piacenza (Pallavicino variety)	Nubia red garlic	Garlic from Voghera	Sulmona red garlic	Garcua white garlic	White garlic from Piacenza (Monticelli variety)	Ail rose de Lautrec PGI
**Acid**	14.2 ± 0.4^bc^	20.5 ± 0.1^e^	14.7 ± 0.0^d^	13.8 ± 0.1^ab^	13.7 ± 0.1^a^	14.5 ± 0.1^cd^	13.5 ± 0.1^a^	14.5 ± 0.1^cd^	20.3 ± 0.0^e^	14.6 ± 0.0^cd^
**Alcohols**	30.6 ± 1.1^d^	31.4 ± 0.3^d^	18.2 ± 0.2^b^	36.8 ± 0.6^e^	23.5 ± 0.2^c^	30.7 ± 0.4^d^	12.7 ± 0.4^a^	24.2 ± 0.1^c^	35.7 ± 0.0^e^	18.9 ± 0.2^b^
**Aldehydes**	40.3 ± 1.0^d^	51.4 ± 0.5^g^	33.9 ± 0.7^b^	56.1 ± 0.2^h^	49.6 ± 0.1^f^	45.8 ± 0.3^e^	32.6 ± 0.3^a^	38.3 ± 0.9^c^	62.1 ± 0.3^i^	32.0 ± 0.6^a^
**Alkanes**	7.1 ± 0.2^ab^	6.9 ± 0.0^ab^	6.6 ± 0.1^a^	13.2 ± 0.4^c^	12.7 ± 0.0^c^	7.4 ± 0.0^b^	6.5 ± 0.0^a^	7.0 ± 0.5^ab^	18.3 ± 0.1^d^	7.1 ± 0.2^ab^
**Alkenes**	32.8 ± 0.7^f^	32.2 ± 0.3^f^	12.9 ± 0.1^a^	31.1 ± 0.4^e^	24.0 ± 0.1^d^	24.4 ± 0.5^d^	17.7 ± 0.3^b^	16.9 ± 0.0^b^	18.8 ± 0.1^c^	17.9 ± 0.2^c^
**Aromatic compounds**	6.4 ± 0.1^b^	nd	6.6 ± 0.1^b^	5.4 ± 0.3^a^	5.7 ± 0.5^a^	12.5 ± 0.2^c^	6.0 ± 0.3^ab^	6.5 ± 0.0^b^	nd	6.0 ± 0.2^ab^
**Ketones**	11.8 ± 0.2^c^	6.0 ± 0.1^b^	nd	5.3 ± 0.1^a^	nd	nd	nd	6.0 ± 0.0^b^	nd	Nd
**Other**	7.0 ± 0.2^a^	12.5 ± 0.9^c^	nd	12.5 ± 0.3^c^	18.0 ± 0.1^d^	7.0 ± 0.1^a^	17.7 ± 0.1^d^	12.9 ± 0.0^c^	11.4 ± 0.1^b^	7.0 ± 0.1^a^
***S*‐alk(en)yl‐l‐cysteine derivates**	214.7 ± 1.4^f^	229.6 ± 1.0^h^	208.8 ± 1.3^e^	174.4 ± 1.2^c^	165.3 ± 1.3^b^	222.4 ± 1.1^g^	152.1 ± 2.3^a^	210.4 ± 1.6^e^	185.3 ± 1.7^d^	223.3 ± 0.5^g^

*Note*: Different letters within a row indicate statistically significant differences among samples (*p* < 0.05). Results are reported as the sum of total area abundance after log_10_ transformation.

Abbreviation: nd, not determined.

Comparative analysis revealed that PDO Polesano white garlic presented the most abundant volatile profile, as reported by both the number (58 features, Table S1) and the abundance (Table 1) of detected volatile compounds. Among the detected classes, *S*‐alk(en)yl‐l‐cysteine sulfoxide derivatives represented the chemical family more abundant in all garlic samples, with PDO Polesano white garlic exhibiting a significantly higher abundance than the other varieties. This finding aligns with previous studies where sulfur compounds were found to be the predominant class of volatile molecules in garlic samples (Kovačević et al. 2023; Yang et al. 2019; Najman et al. 2022; Biancolillo et al. 2022). These compounds are enzymatically derived from alliin, a non‐volatile *S*‐alk(en)yl‐l‐cysteine sulfoxide compound, through the activity of alliinase (EC 4.4.1.4) when plant tissues are damaged (Rose et al. 2005). Specifically, this enzyme converts *S*‐alk(en)yl‐l‐cysteine sulfoxide into pyruvate, ammonia, and sulfenic acids. Formed sulfenic acids further condense to produce thiosulfinates, generating a spectrum of additional sulfur compounds (Keusgen 2011). These compounds are responsible for the flavor and pungency of *Allium* vegetables (Leite et al. 2024), and many are known to be unstable. The second most abundant class of compounds was represented by aldehydes, with their abundance in white garlic samples from Piacenza being significantly higher (*p* < 0.05) than that of other varieties (Table 1). These volatile compounds were also identified as dominant in black garlic (Choi et al. 2017), and most of them comprise carbon chains ranging from six to nine carbons. This is because the enzymes involved in aldehyde production, such as lipoxygenase and hydroperoxide lyase, primarily utilize C6–C9 fatty acids as their substrates. The higher abundance of aldehydes in Piacenza garlic suggests a fresher and less pungent aromatic profile compared with varieties dominated by sulfur compounds. This balance may reflect local agronomic or post‐harvest conditions that preserve lipid‐derived volatiles, contributing to a distinctive sensory identity. In addition, white garlic from Piacenza also exhibited significantly higher (*p* < 0.05) levels of alcohols (Table 1), such as 1‐hexanol (Table S1), which reinforce herbal sensory descriptors, while exhibiting relatively low levels of aromatic compounds compared to other varieties.

**FIGURE 1:**
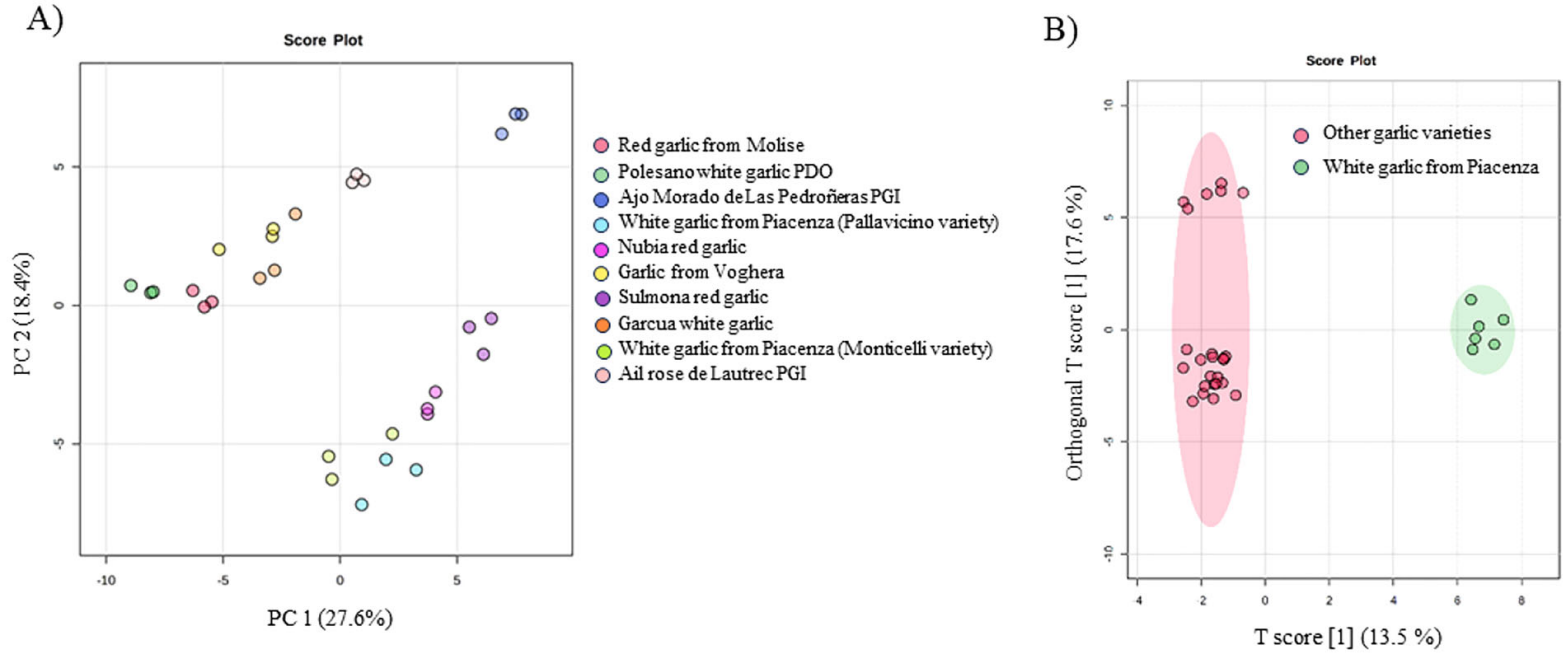
(A) Unsupervised principal component analysis (PCA) score plot of the different volatile molecules detected in garlic varieties. (B) OPLS‐DA score plot distinguishing white garlic from Piacenza (green) from other varieties (pink). Analysis based on log_10_‐transformed volatile abundance data. Each point represents a biological replicate. PDO, protected designation of origin; PGI, protected geographical indication.

To explore whether the volatile composition could effectively discriminate Piacenza white garlic from other varieties, multivariate chemometric analyses were performed on the dataset of identified compounds. First, PCA was performed as a non‐supervised mode of multivariate analysis to investigate natural grouping patterns among samples, and the related score plot is reported in Figure 1A.

In the PCA score plot (Figure 1A), sample clusters showed a clear spatial separation along the first two principal components, which together explained 46% of the total variance. The two varieties of white garlic from Piacenza formed a compact and distinct cluster (positive values of PC1 and negative values of PC2), thus indicating a high degree of similarity in their volatile profiles. This cluster was located close to the Nubia and Sulmona red garlic varieties, suggesting a moderate similarity in their volatile composition. On the contrary, garlic varieties such as Polesano, Garcua, red garlic from Molise, and Voghera clustered farther away (negative values of PC1 and positive values of PC2), as did Ajo Morado de Las Pedroñeras (positive values of PC1 and PC2), reflecting greater differences in their volatile profile. These results indicate that Piacenza garlic possesses a distinctive volatile composition relative to most other varieties, supporting its potential as a unique sensory and chemical profile.

Subsequently, a supervised OPLS‐DA was conducted to highlight the differences between the Piacenza garlic and the other varieties and to identify unique features of white garlic from Piacenza (Figure 1B). The model demonstrated excellent quality, with a prediction ability (Q2) exceeding 80%. Finally, VIP analysis was conducted to interpret the OPLS‐DA model and understand which volatile compounds characterized and discriminated white garlic from Piacenza from the other varieties. The selected volatiles (i.e., those presenting a VIP score >1) are reported in Table 2.

**TABLE 2:** Significant variable importance in projection (VIP) marker molecules that most effectively distinguish the volatilomic profile of white garlic from Piacenza and the other varieties considered in this study.

Volatile compound	VIP[t]	VIP [ortho‐t]	Upregulation in white garlic from Piacenza	Chemical Class	Odor descriptor
Cyclodecane	2.411	0.642	X	Alkane	—
1‐Hexyn‐3‐ol	2.052	0.961	X	Alcohols	—
1‐Methyl‐3‐cyclohexene‐1‐carboxaldehyde	1.985	0.565	X	Aldehydes	—
5‐Hexenal	1.819	0.991	X	Aldehydes	Green
2‐Ethyl‐2‐hexenal	1.744	0.583	X	Aldehydes	Green
1‐Hexanol	1.729	0.618	X	Alcohols	Herbal
1‐Allyl‐3‐(2‐(allylthio)propyl)trisulfane	1.656	0.695		Sulfoxide derivates	Sulfurous
Hexanal	1.590	0.735	X	Aldehydes	Green
(*Z*)‐2‐Methyl‐2‐decene	1.583	0.357	X	Alkenes	
*cis*‐2,3‐Dimethylthiophene	1.500	0.656	X	Sulfoxide derivates	Sulfurous
2,5‐Dimethyl‐2,4‐hexadiene	1.477	0.934	X	Alkenes	—
Decane	1.393	1.013	X	Alkanes	—
2,5‐Dimethylfuran	1.388	1.019	X	Other	Meaty
3‐Ethylcyclohexanone	1.369	1.065	X	Ketone	—
Propyl propanoate	1.369	1.037	X	Acid	Chemical
2‐Methyl‐2‐hexenal	1.337	1.092	X	Aldehydes	Green
5‐Ethyl‐1‐nonene	1.333	0.947	X	Alkenes	—
4‐Methyl‐3‐cyclohexene‐1‐carboxaldehyde	1.317	1.109	X	Aldehydes	—
3‐Methylthiophene	1.316	0.832		Sulfoxide derivates	Sulfurous
1,4‐Cyclohexadiene	1.310	1.113	X	Alkenes	—
6‐Ethyl‐4,5,7‐trithia‐2,8‐decadiene	1.307	1.286		Sulfoxide derivates	—
2‐Ethyl‐trans‐2‐butenal	1.292	0.692		Aldehydes	—
(2*Z*)‐3‐Propyl‐2,4‐pentadien‐1‐ol	1.243	0.758	X	Alcohols	—
Tetrahydro‐2‐methyl‐2*H*‐thiopyran	1.209	1.376		Sulfoxide derivates	—
2,5‐Dimethylthiophene	1.181	0.913		Sulfoxide derivates	Sulfurous
2‐Butenal	1.178	0.486	X	Alkanes	—
2,2,5,5‐Tetramethyl‐3‐cyclopenten‐1‐one	1.174	0.443		Ketone	—
Eugenol	1.089	0.512	X	Alcohols	Spicy
Thiazole	1.071	0.876		Sulfoxide derivates	Fishy
5‐Propyl‐1*H*‐1,2,3,4‐tetrazole	1.047	0.774		Other	—
(*Z*)‐1‐Allyl‐2‐(prop‐1‐en‐1‐yl) disulfane	1.040	0.805	X	Sulfoxide derivates	Sulfurous
2‐Ethyl‐2‐pentenal	1.022	1.116	X	Aldehydes	—

*Note*: Compounds upregulated in white garlic from Piacenza are marked with “X.” VIP[t] and VIP[ortho‐t] refer to scores from OPLS‐DA models. Odor descriptors were retrieved from The Good Scents Company and BenchChem.

Aldehydes and *S*‐alk(en)yl‐l‐cysteine sulfoxide derivates emerged as the primary discriminating classes. According to the VIP scores, a higher accumulation of short‐chain aldehydes was observed in the white garlic variety from Piacenza. Specifically, Piacenza garlic was characterized by a higher accumulation of hexanal, 5‐hexenal, 2‐ethyl‐2‐hexenal, and 2‐methyl‐2‐hexenal, compounds strongly associated with fresh and herbaceous descriptors (Molina‐Calle et al. 2017; Kilic‐Buyukkurt et al. 2023). Their prevalence suggests that Piacenza garlic may deliver a greener, less pungent aroma compared to other varieties. VIP discriminating molecules also comprised alcohol compounds, with 1‐hexyn‐3‐ol, 1‐hexanol, 3‐propyl‐(2*Z*)‐2,4‐pentadien‐1‐ol, and eugenol accumulated in the Piacenza variety. 1‐Hexanol, with its herbal note, is commonly found in fresh garlic (Abe et al. 2020b), whereas eugenol, with its typical spicy descriptor, is typically associated with aged garlic (Abe et al. 2020a). This duality, with green freshness from 1‐hexanol and spicy notes from eugenol, contributes to a more complex aromatic profile than that typically found in fresh garlic varieties. Conversely, *S*‐alk(en)yl‐l‐cysteine sulfoxide derivatives showed lower levels in the Piacenza variety, whereas they emerged as prominent discriminators characterizing the other tested garlic varieties. Among the VIP compounds, (*Z*)‐1‐allyl‐2‐(prop‐1‐en‐1‐yl)disulfane was upregulated in Piacenza garlic and has previously been described as a key constituent of garlic aroma (Song et al. 2023). The prevalence of sulfur compounds is consistent with their recognized importance in garlic, contributing to its characteristic flavor and potential health‐related properties. The general downregulation of this chemical family, together with the upregulation of (*Z*)‐1‐allyl‐2‐(prop‐1‐en‐1‐yl)disulfane, suggests that Piacenza garlic preserves the fundamental garlic aroma while presenting it in a milder and less aggressive form, a feature that may enhance consumer acceptance and market differentiation.

Overall, the VIP analysis highlighted that the volatile profile of Piacenza white garlic is distinguished by a combination of green, herbal, and spicy, supported by moderate levels of some sulfurous volatiles. This distinct composition not only contributes to its sensory uniqueness but also suggests potential as a marker of both geographic origin and quality.

### 3.2 Untargeted Metabolomics Profile of Different Garlic Varieties

To investigate the unique phytochemical fingerprint of Piacenza white garlic, UHPLC–HRMS analysis was conducted across 10 garlic samples, leading to the annotation of 2300 compounds. The MS/MS fragmentation spectra obtained from QC samples facilitated the structural confirmation of a total of 246 compounds. The list of annotated compounds, along with their respective raw abundance values, is provided in Table S2. For each compound, composite mass spectra are also included, including raw intensity data, isotopic distribution patterns, and corresponding MS/MS fragmentation spectra. The annotated metabolites comprise 270 organosulfur compounds (of which 21 are specific to garlic), 264 polyphenols (including 24 anthocyanins, 18 lignans, 2 flavonols, 60 flavones, 6 flavanols, 98 low molecular weight phenolics, 49 phenolic acids, and 7 stilbenes), 38 glucosinolates, and 24 carotenoids. The results align with the typical composition reported for garlic extracts obtained by extracting cloves with hydroalcoholic solutions, which concentrate water‐soluble organic compounds (Shang et al. 2019; Martins et al. 2016). Garlic ranks among the vegetables with the highest total phenolic content (Lanzotti et al. 2014). Additionally, organosulfur compounds are key molecules in defining garlic's characteristic flavor and being responsible for many of its health benefits (Ramaaa et al. 2006). Indeed, these molecules are highly interesting in human nutrition and health because they have proven antioxidant (Boonpeng et al. 2014), cardiovascular protective (Ba et al. 2012), anticancer, anti‐inflammatory (Arreola et al. 2015), immunomodulatory (Percival 2016), and antibacterial (Bhatwalkar et al. 2021) properties. The mechanical disruption of garlic cloves triggers enzymatic reactions that swiftly generate a wide array of sulfur compounds (Lanzotti et al. 2014). Thiosulfinates, including allin, cannot be detected due to their high degradability in liquid chromatography, whereas sulfides and γ‐glutamyl‐l‐cysteine catabolic metabolites are then available (Amagase 2006). In UHPLC–HRMS analysis, considering Table S2, the presence of dimethyl disulfide, divinyl sulfide, methylpropenyl tetrasulfide, *S*‐methylcysteine sulfoxide, *S*‐allylmercaptocysteine, and *N*‐acetyl‐*S*‐allylcysteine, as well as some precursors such as γ‐glutamyl‐l‐cysteine and l‐γ‐glutamyl‐*S*‐allylthio‐l‐cysteine, which are intermediates in *S*‐allyl‐l‐cysteine sulfoxide's biosynthesis beside being part of the cell nitrogen and sulfur storage pool (Jabbes et al. 2012; Martins et al. 2016), proved the typical garlic composition.

**FIGURE 2:**
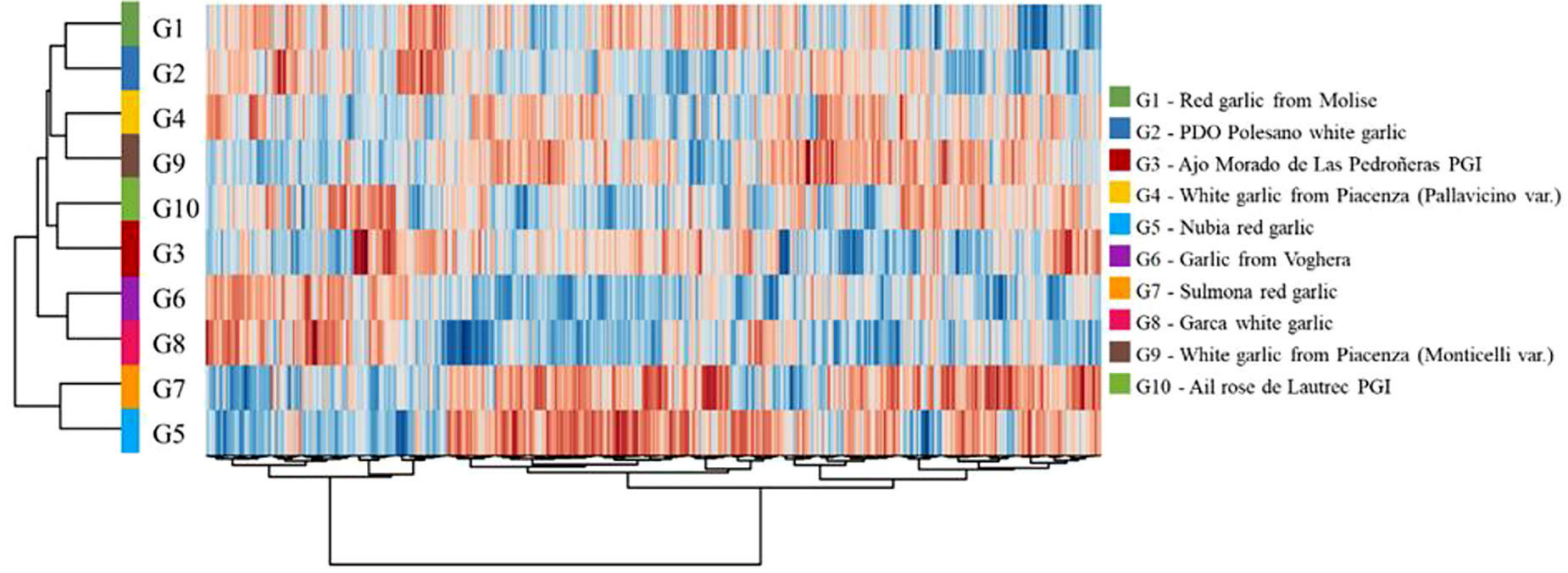
Unsupervised hierarchical cluster analysis (HCA) of the metabolic compounds identified in garlic varieties through UHPLC–HRMS. Rows = garlic sample metabolites; columns = metabolites. The two white garlic varieties from Piacenza are closely clustered. PDO, protected designation of origin; PGI, protected geographical indication.

An unsupervised HCA based on the FCs of each metabolite was used to evaluate the fingerprint of the two samples of white garlic from Piacenza compared to the other garlic varieties (Figure 2).

The model identified two main clusters, one comprising only the Nubia (G5) and Sulmona (G7) red garlic varieties. At the same time, the second cluster grouped all the other samples, including the two varieties of garlic from Piacenza (G4 and G9), closely related to each other, as well as the red garlic from Molise (G1) and the Polesano garlic PDO (G2). The same clustering pattern was observed in the volatilome dataset when subjected to PCA (Figure 1A), indicating consistency in the sample clustering based on chemical composition and volatile profile. The distinct profile of white garlic from Piacenza was also previously observed by Ritota et al. (2012) through multivariate analysis of ^1^H HRMAS‐NMR spectra, although the findings were based on a limited coverage of metabolites, likely due to the different analytic technique.

**FIGURE 3:**
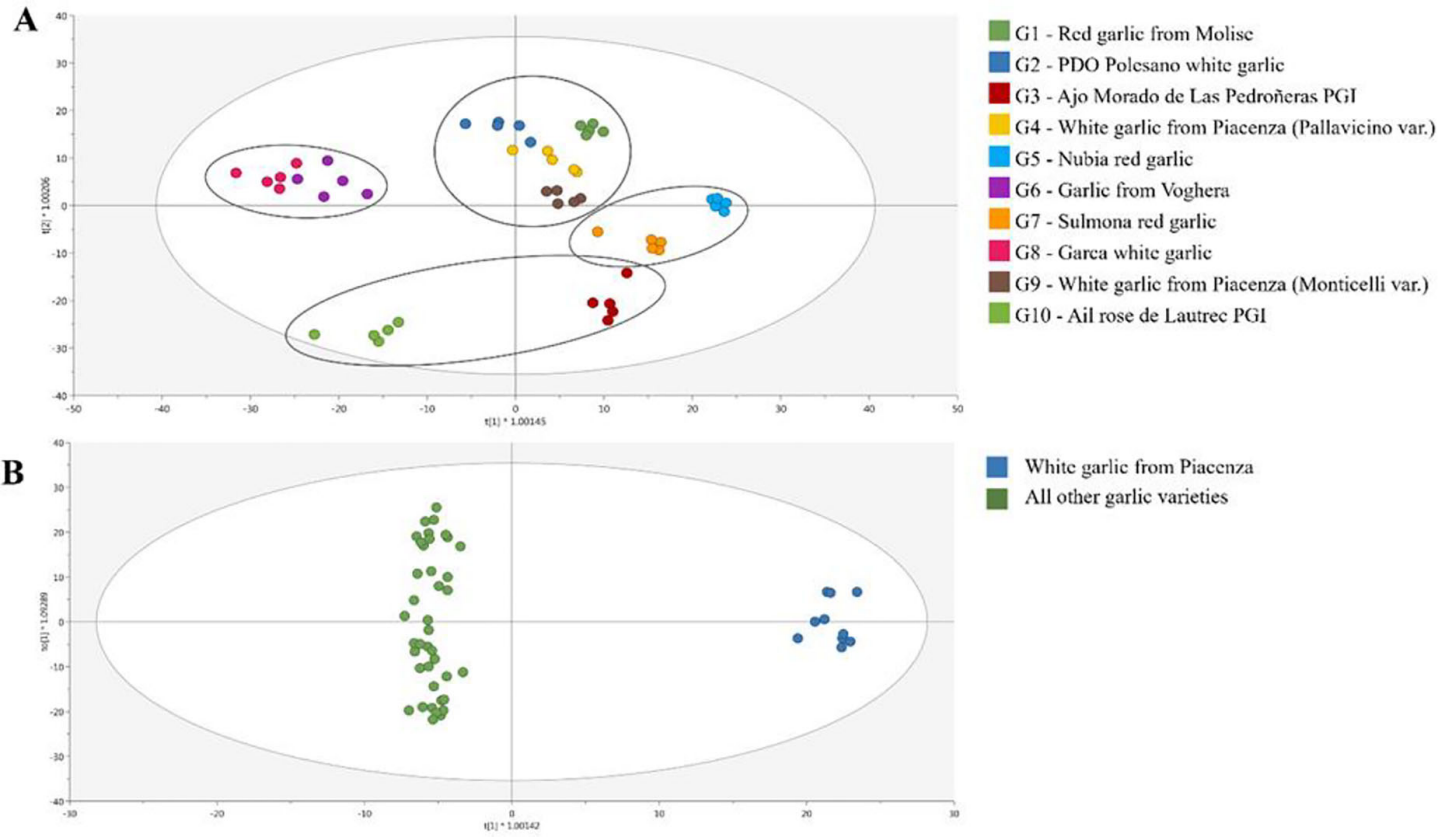
Orthogonal projection to latent structures discriminant analysis based on (A) the 10 garlic varieties analyzed in the study and (B) the white garlic from Piacenza samples (blue cluster) and the other varieties considered in this study taken together (green cluster). PDO, protected designation of origin; PGI, protected geographical indication.

The supervised OPLS‐DA modeling approach highlighted the compounds responsible for differentiating the 10 garlic groups. The resulting model (Figure 3A) showed high goodness of fitting (*R* ^2^ *Y* = 0.955) and prediction ability (*Q* ^2^ *Y* = 0.818).

The outlined results confirmed the HCA clusterization. The VIPs with a score >1.2 were selected, reporting 504 compounds having higher discrimination potential across all the garlic samples (Table S3). The most representative classes identified within the VIPs were amino acids and derivatives such as dipeptides, oligopeptides, and polyphenols. Histidinyl‐asparagine, glycyl‐arginine, asparaginyl–tyrosine, and cysteinyl–tyrosine were the compounds with higher discriminating potential within amino acids and derivatives, whereas polyphenols included delphinidin, isorhamnetin 3‐(6″‐malonylglucoside), and feruloyltartaric acid. Considering this model and the huge number of variables considered, it is possible to assume that the highlighted compounds are related to the characteristics of the individual garlic species analyzed. Cho et al. (2009) reported that the color of the garlic clove was related to the specific ratio of dipeptides with the S group, whereas Chen et al. (2013) brought experimental evidence that 43 different garlic varieties had different polyphenol profiles. Similar observations were made in elephant garlic, where dipeptides, sulfur metabolites, and glutathione were identified as varietal markers for authentication purposes (Carullo et al. 2024). Being mainly interested in investigating the differences underlying the two varieties of white garlic from Piacenza compared to all the other garlic samples taken together, despite the different geographical origins, a second OPLS‐DA model was then performed, thus reducing the number of variables (Figure 3B). The model proved high *R* ^2^ *Y* (0.993) and *Q* ^2^ *Y* (0.907) values, together with a significant validation from CV‐ANOVA (*p* = 1.42 × 10^−27^ for regression). Based on the model parameters, there is an effective separation between the two groups due to the annotated metabolites. The VIPs (score > 1.2) calculated corresponded to 496 compounds, which mainly included organosulfur compounds (78), organooxygen compounds (70), and flavonoids (55). The remaining metabolites belonged to different chemical classes, including, among others, steroids, benzopyranes, phenolic lipids, and glycerophospholipids. The whole list of compounds differentiating white garlic from Piacenza from the other samples is reported in Table S4. The potential of these compounds to distinguish the white garlic from Piacenza from the others was then examined by calculating an ROC curve, and the corresponding AUC values were evaluated (Table S4). In many different works (Rivera‐Pérez et al. 2023; Senizza et al. 2019), the area under the ROC curve (AUC) was employed as an indicator of statistical robustness, reflecting the discriminatory power of each compound selected on the basis of VIP scores between the two comparison groups. Considering the 64 compounds with a VIP score >2 (Table S4), 51 out of 64 (80% of the total) had an AUC value between 0.8 and 1, which can be considered best classifiers, as reported in the literature (Xia et al. 2013); however, only 13 compounds had a score lower than 0.8 or were not validated by the ROC curve. Such high AUC values underline the robustness of the metabolomic fingerprint for varietal traceability, as already demonstrated in Italian garlic through HRMAS‐NMR multivariate approaches (Ritota et al. 2012) and in more recent rapid PS–MS metabolomics studies applied to garlic of different origins (Leite et al. 2024). The compounds with the higher VIP and ROC values are reported in Table 3.

**TABLE 3:** Compounds identify as marker in white garlic of Piacenza with variable importance in projection (VIP) score >2.5 and area under the receiver operating characteristic curve (AUC) >0.9 through ultrahigh‐performance liquid chromatography coupled with high‐resolution mass spectrometry (UHPLC–HRMS).

Common name	Chemical class	VIP score	AUC
Melledonal C	Sesquiterpenes	2.90883	1
LysoPE (0:0/16:0)	Lipid	2.85064	0.975
5‐Hydroxyindole thiazolidine carboxylate	S‐containing molecules	2.82367	0.995
Lipoyl‐GMP	S‐containing molecules	2.80663	0.9825
Pyro‐l‐glutaminyl‐l‐glutamine	Amino acids and derivates	2.72613	0.9825
1‐Isobutanol	Glycoside	2.7169	1
Triethyl citrate	Carbonyl compounds	2.68255	0.99
Demethylcalabaxanthone	Xanthones	2.65281	0.98
(*S*)‐Cajaflavanone	Flavonoids	2.6388	0.995
2,5‐Diamino‐6‐(5‐phospho‐d‐ribosylamino)pyrimidin‐4(3*H*)‐one	*N*‐Glycosyl compounds	2.6228	0.985
Glucosyringic acid	Benzoates	2.61196	0.9775
Physalin E	Secosteroids	2.61051	1
2‐Oxoarginine	Carboxylic acid	2.5932	1
(*R*)‐Bitalin A	Acetophenones	2.57585	0.9825
Poncitrin	Coumarins	2.57512	0.955

The most representative classes identified within the VIPs were carboxylic acids and derivates, in particular amino acids and derivates, such as dipeptides and oligopeptides, such as l‐alloisoleucine, N2‐fructopyranosylarginine, and *N*6‐acetyl‐5*S*‐hydroxy‐l‐lysine. Flavonoids, including poncitrine and (*S*)‐cajaflavone, were identified. The class of organosulfur compounds was associated with S‐containing molecules such as 5‐hydroxyindole thiazolidine carboxylate, lipoyl–GMP, and cysteinyl–tyrosine. Regarding organooxygen compounds and, in particular, carbohydrates and derivatives, pyro‐l‐glutaminyl‐l‐glutamine, 1‐isobutanol, and 2,5‐diamino‐6‐(5‐phospho‐d‐ribosylamino)pyrimidin‐4(3*H*)‐one were observed. Both OPLS‐DA models showed the same classes of discriminating compounds, although with differences in the highlighted compounds.

**FIGURE 4:**
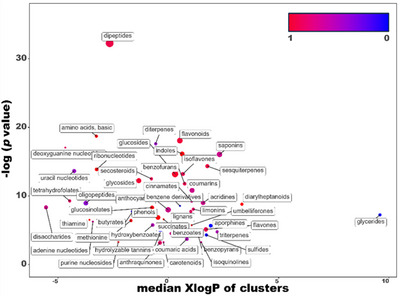
Chemical similarity enrichment analysis (ChemRICH) of VIP compounds >1.2 resulting from OPLS‐DA supervised model based on garlic from Piacenza compared to all the other samples taken together. The *X*‐axis indicates the median XlogPs of clusters, whereas the *Y*‐axis refers to the −log(*p* value), indicating the statistical significance of clusters based on the Kolmogorov–Smirnov test. Each cluster corresponds to a significantly altered group of metabolites, whereas its size provides information about the number of compounds included. The ratio scales reflect the relative abundance of metabolites either up‐accumulated (red) or down‐accumulated (blue) across clusters. An interactive version of the plot is available at the link: https://chemrich.fiehnlab.ucdavis.edu/ocpu/tmp/x0e06809460/files/ggplotly.html.

To enhance the discrimination between Piacenza's white garlic and all other varieties, a statistical enrichment strategy was employed to examine differences in metabolite composition. To this end, a ChemRICH enrichment analysis was performed on VIP molecules (threshold score >1.2); the resulting plot is shown in Figure 4, whereas the comprehensive list of significant compounds is available in Table S5.

In general, a wide range of polyphenols (xanthones, flavonoids, isoflavones, flavones, lignans, stilbenes, and phenolic glycosides) were spotted as clusters significantly up‐accumulated in the garlic from Piacenza compared to other locations, whereas phenols (catechols, hydrolyzable tannins, coumaric acid, and hydroxybenzoates) were found to be down‐accumulated. Concerning the organosulfur compounds composition, the results (Figure 4, Table S5) indicated that glucosinolates and methionine (such as *S*‐methylcysteine sulfoxide and deoxyalliin) were predominantly accumulated in Piacenza's varieties, whereas all the other varieties, on average, accumulated sulfur compounds, sulfides, and butyrates. The enrichment analyses also pointed out the up‐accumulation of dipeptides and the down‐accumulation of amino acids and oligopeptides in Piacenza's garlic compared to the others. These compositional trends are consistent with the hypothesis that genotype × environment interactions drive the metabolic fingerprint of garlic, as previously shown for organosulfur and phenolic compounds in different growing conditions (Barboza et al. 2022; Pacholczyk‐Sienicka et al. 2024). Finally, despite being significant in determining the garlic profile (Shang et al. 2019; Martins et al. 2016), other groups of molecules, such as saponins, disaccharides, anthocyanins, and coumarins, showed no trend between the two groups. The different abundance in Piacenza garlic compounds’ composition compared to the average of the others could be due to the different processing times, as the samples were acquired from the local markets, and no clear indication of the harvesting time was present (Molina‐Calle et al. 2017), but also to its genotype (Hirata et al. 2016; Khar et al. 2011). Accordingly, besides the well‐known typical molecules of garlic composition, quantification of amino acids, dipeptides, and oligopeptides can be used as a tool for characterizing the different varieties as suggested by the specific up‐ and down‐regulation of these compounds among species and varieties (Soininen et al. 2014). From a broader perspective, the observed metabolomic differences may also translate into distinctive sensory and nutraceutical properties, as variations in sulfur and polyphenolic compounds are known to influence garlic flavor intensity and potential health‐promoting effects (Jain et al. 2025). These overall refer to the “terroir” effect, known as the interlink between the genetic background and the geographical origin (e.g., climate, soil). Indeed, as already discussed by Lucini et al. (2020), the modulation of phytochemical profiles in plant‐based foods strictly depends on the terroir, which thus marks the differences in products’ chemical composition.

### 3.3 Data Integration Between Volatilomics and Metabolomics

**FIGURE 5:**
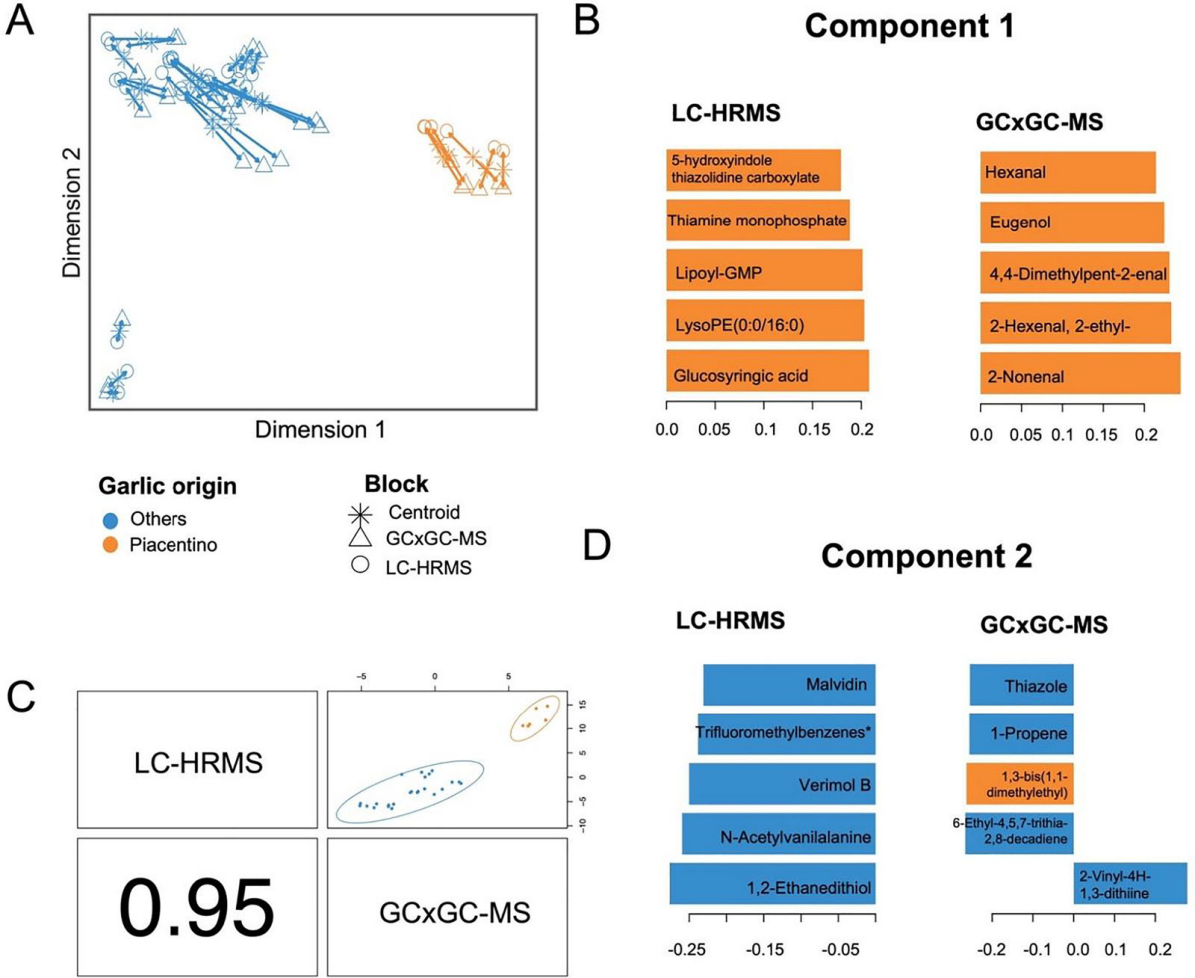
Data integration of UHPLC–HRMS and SPME–GC × GC–MS metabolic profiles datasets on the discrimination between Piacenza's white garlic and other varieties. (A) Arrow plot of different sample block integration projected into the first two predicting components. (B and D) Loading plots highlighting the key metabolites most strongly associated with the origin factor for both data blocks (UHPLC–HRMS and SPME–GC × GC–MS), based on the previously optimized components: Component 1 and Component 2, respectively. (C) Pearson's correlation score between the two blocks considering the first component. The specific name of this metabolite is alpha‐(Methoxyimino)‐*N*‐methyl‐2‐[[[1‐[3‐(trifluoromethyl)phenyl]ethoxy]imino]methyl]benzeneacetamide. GC, gas chromatography; HRMS, high‐resolution mass spectrometry; LC, liquid chromatography; MS, mass spectrometry.

Data integration using a supervised DIABLO framework was conducted to establish correlations between metabolic insights obtained from SPME–GC × GC–MS (volatile metabolites) and UHPLC–HRMS (non‐volatile metabolites) datasets. The resulting model is illustrated in Figure 5.

First, an arrow plot of all samples was projected onto the first two dimensions to investigate the discrimination potential of garlic origin (Piacentino vs. others) and to assess the correlation degree between the samples from the two datasets (Figure 5A). The combined contribution of the two omics approaches clearly demonstrated the discrimination capacity in distinguishing Piacenza's white garlic from other varieties. Indeed, the two datasets were characterized by a low spatial distance, as indicated by the length of the arrows connecting them, which suggests a high correlation. This finding was further supported by high Pearson's correlation coefficients (*r* = 0.95) between selected features from both datasets, as shown in Figure 5C.

The most discriminant metabolites were then selected from the model, which highly contributed to the insight among Piacenza and other garlic samples analyzed. These compounds, associated with each dataset and for the two considered components, are depicted in Figure 5B,D for Components 1 and 2, respectively. Furthermore, a comprehensive list of metabolites is provided in Table S6. Specifically, features isolated from the first component were attributed to the discrimination of Piacenza's white garlic, as indicated in the loading plot (Figure 5B), which identified the most important metabolites that contributed positively to the discrimination of Piacentino garlic in the integration model. In contrast, the second component was exclusively implicated in distinguishing other garlic varieties (Figure 5D). The resulting model highlighted several classes of compounds as most discriminant for Piacenza's garlic samples (Table S6), including tannins (glucosyringic acid), lipids and sterols (i.e., lysoPE (0:0/16:0), lysoPC (16:0), physalin‐E, ‐Q, and ‐A), amino acids and derivatives (i.e., 5‐hydroxyindole thiazolidine carboxylate and N2‐fructopyranosylarginine), and polyphenols (i.e., poncitrin, (*S*)‐cajaflavanone, 2″,4″,6″‐triacetylglycitin, and apigenidin), as well as other classes such as terpenoids, organosulfur, and alcohol compounds. Piacentino garlic represents a good health‐promoting source compared to other analyzed garlics, due to the specific content of polyphenolic compounds, as reported as discriminant markers in the data integration model, including flavonoids and organosulfur compounds. El‐Saadony et al. (2024) associated these compounds contained in garlic with their principal medicinal properties, including antimicrobial and antioxidant activities. In fact, Gu et al. (2019) confirmed the antioxidant capacity of tannins found in garlic, which is notable when examining different food matrices. Interestingly, the presence of lipids and sterols as biomarkers of Piacentino garlic confirms the findings of Molino et al. (2021), who determined several lipid classes in a Philippine garlic cultivar and suggested the use of these specific metabolite groups to serve as a basis for comparing different cultivars. Indeed, lipids supply essential fatty acids and enhance the texture and mouthfeel of food, as well as acting as Maillard precursors, which intensify flavor and aroma together with amino acids and sugars contained in the garlic (Shahidi 2000).

Considering volatile compounds characterizing Piacenza's white garlic, both sulfur and non‐sulfur compounds were reported. Piacenza's white garlic samples were primarily discriminated by the presence of non‐sulfur molecules such as aldehydes (i.e., 2‐nonenal, 2‐hexenal, 4,4‐dimethylpent‐2‐enal, 2‐octenal, and 2‐heptenal), followed by ketones and alcohols. It is noteworthy that sulfide compounds such as (*E*)‐1‐methyl‐2‐(prop‐1‐en‐1‐yl)disulfane, diallyl disulfide, (*E*)‐allyl(prop‐1‐en‐1‐yl)sulfane, allyl mercaptan, (*Z*)‐allyl(prop‐1‐en‐1‐yl)sulfane, and dimethyl trisulfide were reported to be highly specific to Piacenza's white garlic. Conversely, other garlic varieties were mainly represented by the high presence of alkyl thiosulfates (i.e., 1,2‐ethanedithiol, methyl methanethiosulfonate, and *S*‐methyl methanesulfinothioate), benzene and substituted derivatives (i.e., verimol B), and organooxygen compounds (i.e., triglochinin and 6‐methylthiohexyldesulfoglucosinolate). Furthermore, they were discriminated by the presence of 6‐ethyl‐4,5,7‐trithia‐2,8‐decadiene, benzene, 1‐propene, thiazole, and 2‐ethyl[1,3]dithiane. Moreover, a few sulfur‐containing compounds were reported to be slightly abundant in these varieties, including 1‐allyl‐3‐(2‐(allylthio)propyl)trisulfane, (*E*)‐allyl(prop‐1‐en‐1‐yl)sulfane, and sulfide, allyl methyl, and (*Z*)‐1‐Allyl‐2‐(prop‐1‐en‐1‐yl)disulfane. As discussed earlier, some of the discriminating compounds associated with the characterization of Piacenza's white garlic samples were already featured by the OPLS‐DA supervised models, underscoring first of all the presence of aldehydes and alcohols in Piacentino garlic as an index of fresh, green, and herbal sensory notes. Notably, the enrichment of (*Z*)‐1‐allyl‐2‐(prop‐1‐en‐1‐yl)disulfane indicates that Piacenza garlic maintains the essential garlic aroma in a softer, less pungent expression (Song et al. 2023). The importance of various compounds found in Piacenza's garlic varieties, including polyphenols, sterols, sulfur‐containing compounds, carbohydrates, oligopeptides, and amino acids, as well as aldehydes, ketones, and alcohols, collectively contribute to the wide range of garlic's applications, which include cholesterol reduction, cardiovascular risk mitigation, immune system enhancement, cancer prevention, and overall improvement of heart health (Tudu et al. 2022). Moreover, the presence of aromatic and sulfur‐containing metabolites as well as amino acids and derivatives, including peptides, in Piacenza's white garlic varieties confirmed the finding reported by several authors of using amino acid content as biomarkers for garlic geographical discrimination (Ritota et al. 2012; Jo et al. 2020; Mi et al. 2021; Carullo et al. 2024).

To resume, the multiblock analysis integrating LC–HRMS and GC × GC–MS data effectively discriminated Piacenza garlic from other varieties. The clear separation observed along the first component indicates that both volatile and non‐volatile metabolite profiles contribute significantly to the varietal distinction. Key discriminant compounds included sulfur‐containing volatiles such as 2‐nonenal, hexanal, and eugenol, as well as non‐volatile metabolites like thiamine monophosphate and glucosyringic acid. The high correlation between data blocks (*r* = 0.95) confirms the complementarity and robustness of the integrated approach. Overall, the results support the identification of potential varietal markers for Piacenza garlic and provide a reliable basis for subsequent supervised modeling.

### 3.4 Limitations of the Study

As a case study, the work presents some limitations that should be considered:

- Sample size and biological replicates. Only five bulbs per variety were analyzed, which may have affected the robustness of the multivariate analysis and the generalizability of the findings. In foodomics studies, the use of larger and more representative sample sets is generally recommended to improve model performance and reduce the impact of analytical and biological variability. Although cross‐validation or resampling procedures can be used when sample availability is limited, small sample sizes may still lead to misclassification rates and false positives, complicating the interpretation of whether the observed differences are statistically significant (Oliveri 2017).
- Post‐harvest and storage conditions. Sample collection was performed without strict control over post‐harvest handling, storage, or harvesting time. Variations in these factors could have influenced the observed chemical profiles and introduced uncontrolled variability. In plant‐based foodomics studies, it is well established that post‐harvest conditions, such as temperature, humidity, and storage duration, can significantly affect metabolite stability and composition (Yun et al. 2022). Differences in these parameters may lead to degradation, enzymatic transformation, or shifts in secondary metabolites, potentially confounding the interpretation of results. Therefore, the lack of uniform post‐harvest control represents an important source of experimental variability that should be considered.
- Incomplete information on agronomic and environmental factors. Detailed data on genotype, agronomic practices, and pedoclimatic conditions were not uniformly available for all varieties. This limitation prevents a clear attribution of observed chemical differences to specific factors such as genetics, environment, or cultivation system. Furthermore, seasonal variability and climate‐related changes can introduce additional shifts in chemical composition, which underscores the importance of standardized and well‐documented sampling protocols.

Nonetheless, the primary aim of this work was to develop an untargeted foodomics‐based fingerprinting approach capable of capturing global chemical differences among garlic varieties and identifying discriminant compounds, particularly those associated with Piacenza white garlic.

## 4 Conclusion

In this study, foodomics methodologies, based on metabolomic and volatilome profiling, were employed to comprehensively profile the phytochemical and volatile compositions of different varieties of garlic. As a case study, white garlic from Piacenza, a typical Italian agri‐food product produced in the province of Piacenza and recognized by the Emilia Romagna Region, was investigated to highlight its distinctiveness from other European garlic varieties. For the first time, the integration of metabolomic and volatilomic datasets was applied to identify potential discriminant compound characteristics of the Piacenza variety. Overall, 89 volatile and more than 2300 non‐volatile compounds were annotated, revealing that Piacenza garlic possesses unique chemical signatures. It was characterized by a higher accumulation of short‐chain aldehydes and alcohols, which confer fresh, green, and herbal sensory notes, while maintaining moderate levels of sulfur volatiles. Among these, the enrichment of (*Z*)‐1‐allyl‐2‐(prop‐1‐en‐1‐yl)disulfane suggested that Piacenza garlic preserves the fundamental garlic aroma in a milder and less aggressive form. At the same time, the upregulation of polyphenols, flavonoids, and dipeptides, together with unique enrichment patterns of glucosinolates and amino acid derivatives, further contributed to its distinct metabolic profile and highlighted the interplay between genetic background and terroir. These findings support the recognition of Piacenza garlic as a product with a unique sensory and chemical identity, which may serve as a marker of both origin and quality.

Some limitations should be acknowledged, such as detailed information on genotype and agronomic practices and the sample size and collection, which could have influenced the observed chemical profiles. For these reasons, future studies should aim to validate the proposed markers by including a broader sampling strategy and considerations regarding genotypic data and sensory evaluation to strengthen the link between chemical markers and perceived quality attributes.

Nonetheless, this work confirms the potential of combining orthogonal analytical approaches, such as volatilomics and metabolomics, coupled with advanced data management approaches, to provide comprehensive chemical signatures of foods. The proposed foodomics approach may find interesting applications in the integrity and quality aspects of food science and contributes to the valorization of typical agri‐food products such as Piacenza white garlic, reinforcing their quality identity.

## Author Contributions

**Giulia Leni**: formal analysis, investigation, methodology, data curation, software, writing – original draft, writing – review and editing. **Marco Armando De Gregorio**: formal analysis, investigation, data curation, software, writing – original draft, writing – review and editing. **Elena Secomandi**: formal analysis, investigation, data curation, software, writing – original draft, writing – review and editing. **Leilei Zhang**: investigation, data curation, software, writing – original draft, writing – review and editing, formal analysis. **Terenzio Bertuzzi**: methodology, resources, supervision, writing – review and editing, funding acquisition. **Marco Trevisan**: resources, supervision, writing – review and editing, project administration, funding acquisition. **Giorgia Spigno**: resources, writing – review and editing, funding acquisition. **Luigi Lucini**: methodology, resources, conceptualization, supervision, writing – review and editing, software, funding acquisition.

## Conflicts of Interest

The authors declare no conflicts of interest.

## Supporting information




**Supporting Table1**: Comprehensive list of putatively volatile compounds identified in garlic samples.
**Supporting Table2**: Comprehensive list of tentatively annotated compounds identified in garlic cloves using UHPLC–Orbitrap–HRMS.
**Supporting Table3**: Comprehensive list of key discriminant metabolites (VIP score > 1.2) obtained from supervised OPLS‐DA of the metabolites identified in the different garlic varieties analyzed.
**Supporting Table4**: Comprehensive list of discriminant metabolites (VIP score > 1.2) resulting from
**Supporting Table5**: List of significantly altered metabolite clusters in garlic from Piacenza compared to all the other varieties taken together.
